# Safe and Stable Lithium Metal Batteries Enabled by an Amide-Based Electrolyte

**DOI:** 10.1007/s40820-021-00780-7

**Published:** 2022-01-12

**Authors:** Wanbao Wu, Yiyang Bo, Deping Li, Yihong Liang, Jichuan Zhang, Miaomiao Cao, Ruitian Guo, Zhenye Zhu, Lijie Ci, Mingyu Li, Jiaheng Zhang

**Affiliations:** 1 Sauvage Laboratory for Smart Materials, Harbin Institute of Technology (Shenzhen), Shenzhen, 518055 People’s Republic of China; 2 Research Centre of Printed Flexible Electronics, School of Materials Science and Engineering, Harbin Institute of Technology (Shenzhen), Shenzhen, 518055 People’s Republic of China; 3 School of Materials Science and Engineering, Harbin Institute of Technology (Shenzhen), Shenzhen, 518055 People’s Republic of China; 4 Department of Chemistry, University of Idaho, Moscow, ID 83844-2343 USA

**Keywords:** Amide-based electrolyte, Nonflammable, Inorganic/organic-rich solid electrolyte interphase, Dendrite-free, Lithium metal batteries

## Abstract

**Highlights:**

- A novel amide-based nonflammable electrolyte is proposed. The formation mechanism and solvation chemistry are investigated by molecular dynamics simulations and density functional theory.
- An inorganic/organic-rich solid electrolyte interphase with an abundance of LiF, Li_3_N and Li–N–C is in situ formed, leading to spherical lithium deposition.
- The amide-based electrolyte can enable stable cycling performance at room temperature and 60 ℃.

**Abstract:**

The formation of lithium dendrites and the safety hazards arising from flammable liquid electrolytes have seriously hindered the development of high-energy-density lithium metal batteries. Herein, an emerging amide-based electrolyte is proposed, containing LiTFSI and butyrolactam in different molar ratios. 1,1,2,2-Tetrafluoroethyl-2,2,3,3-tetrafluoropropylether and fluoroethylene carbonate are introduced into the amide-based electrolyte as counter solvent and additives. The well-designed amide-based electrolyte possesses nonflammability, high ionic conductivity, high thermal stability and electrochemical stability (> 4.7 V). Besides, an inorganic/organic-rich solid electrolyte interphase with an abundance of LiF, Li_3_N and Li–N–C is in situ formed, leading to spherical lithium deposition. The formation mechanism and solvation chemistry of amide-based electrolyte are further investigated by molecular dynamics simulations and density functional theory. When applied in Li metal batteries with LiFePO_4_ and LiMn_2_O_4_ cathode, the amide-based electrolyte can enable stable cycling performance at room temperature and 60 ℃. This study provides a new insight into the development of amide-based electrolytes for lithium metal batteries.
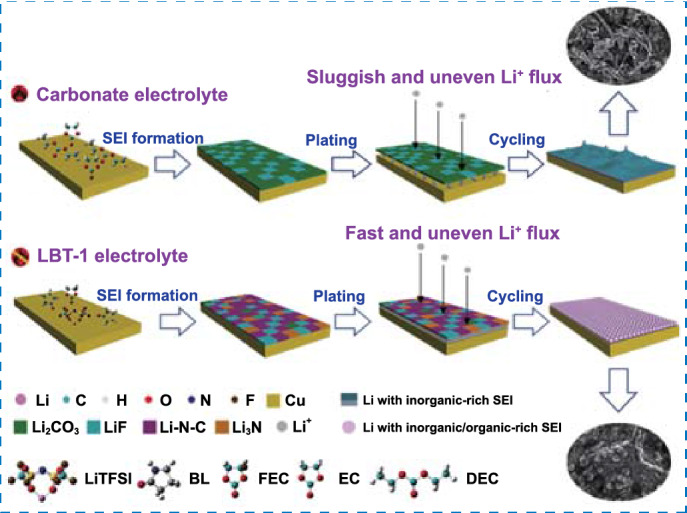

**Supplementary Information:**

The online version contains supplementary material available at 10.1007/s40820-021-00780-7.

## Introduction

As fossil fuels continue to be depleted, the development of sustainable new energy is of great significance for social development [1, 2]. Lithium-ion battery technology has already proved its value in the 2019 Nobel Prize. However, to further expedite the application of EVs and reduce the usage of fossil fuels, commercial lithium-ion batteries having graphite anode still need improvements in energy density. Therefore, it is urgent to develop next-generation high-energy–density batteries [3]. Lithium metal has become one of the most promising anode materials, owing to its ultrahigh specific capacity (3860 mAh g^−1^) and lowest electrochemical potential (− 3.04 V vs. standard hydrogen electrode) [4, 5]. Despite these advantages, the development of practical lithium metal battery (LMB) is still hampered by issues like by low coulomb efficiency, Li dendrite growth and unstable solid electrolyte interface (SEI) layers [6–8]. The growth of Li dendrites can reduce coulomb efficiency and trigger short circuits within the cell, resulting in serious safety hazards [9, 10]. Lithium metal can react with the electrolyte to form an SEI layer on the interface. A stable SEI layer could prevent further decomposition of the electrolyte, while some an unstable SEI layer consumes the electrolyte during the battery cycling. Therefore, it is crucial to construct a stable SEI layer toward a superior battery performance [7, 11, 12].

Numerous strategies, such as artificial SEI structures, protective coating and electrolyte engineering (manipulating Li salts, solvent or additives), have been developed to regulate the electrochemical deposition of lithium [13–22]. Although artificial SEI design and protective coating can effectively inhibit the growth of Li dendrites, the tedious fabrication procedure and the drastically increased interfacial impedance originating from the formation of non-smooth thick layers further limit the practical prospect [23–26]. In comparison, optimizing the electrolyte becomes the most promising and facile approach owing to its easy operation for scale-up and low cost. For example, it has been reported that electrolyte engineering can enable the formation of Li_3_N layer and incorporation of Li–N–C groups on the surface of Li metal, which can serve as the Li-ion diffusion channels and inhibit Li dendrite growth [27–30].

As for lithium metal batteries, the commonly used electrolytes can be divided as carbonate-based electrolytes and ether-based electrolytes. However, carbonates have the disadvantages of flammability, volatility, poor thermal stability and easily causing lithium dendrites growth, while the low electrochemical oxidation potential of ethers limits their practical application [23, 31]. Therefore, it is urgent to develop a safe electrolyte having characteristics like nonflammability, preventing formation of lithium dendrites and good interfacial compatibility with lithium metal anode. Numerous works have been carried out to develop such a safe electrolyte. The researchers not only tried optimizing the conventional electrolytes, but also proposed various new concepts including super-concentration electrolyte [32], ionic liquids [33], polymer electrolyte [34] and solid-state electrolyte [35]. Among those proposed strategies, liquid electrolytes can have better wettability with electrode materials and greater ion transport ability. Therefore, it is desirable to design a new liquid electrolyte by electrolyte engineering, which can, at the same time, solve the issues of flammability and lithium dendrite growth in conventional electrolytes.

In this work, we propose a novel amide-based electrolyte composed of LiTFSI and butyrolactam (BL), which can keep a liquid state at room temperature. Meanwhile, we employed 1,1,2,2-tetrafluoroethyl-2,2,3,3-tetrafluoropropylether (TTE) as a co-solvent to reduce the viscosity and improve the compatibility with the lithium metal. Besides, fluoroethylene carbonate (FEC) was introduced as an additive to further stabilize the interfaces. With the above delicately designed electrolyte, a stable SEI layer with inorganic/organic-rich components (LiF, Li_3_N and Li–N–C) can be in situ formed, which can effectively regulate the morphology and structure of lithium to achieve its uniform deposition. The formation mechanism of the amide-based electrolyte and its ionic morphology are further elucidated by density functional theory (DFT) and molecular dynamics simulations (MD simulations). The amide-based electrolyte shows good compatibility with lithium metal and exhibits superior cycling performance in Li||LFP and Li||LMO full cells. Moreover, the electrolyte can resist high temperatures (60 °C) and guarantee stable operation of batteries. The carefully designed amide-based electrolyte can also be extended to zinc-ion batteries and other metal-ion batteries. There is no doubt that this work can broaden the pathways for the future electrolyte development.

## Experimental Section

### Electrolyte Preparation

Lithium bis(trifluoromethanesulfonyl)imide (LiTFSI), butyrolactam (BL) and fluoroethylene carbonate (FEC) were purchased from Aladdin Reagent Co., Ltd. 1,1,2,2-Tetrafluoroethyl-2,2,3,3-tetrafluoropropylether (TTE) was provided by Dadao New Material Technology Co., Ltd.

The electrolytes were prepared by mixing different molar ratios of LiTFSI and BL with 5 wt% FEC at room temperature until a transparent solution was obtained. The obtained electrolyte was denoted as LB-x (x indicates the molar ratio of LiTFSI to BL is 1:x). Similarly, LBT electrolyte was prepared by mixing TTE and LB-3 electrolyte with various molar ratios, and the final electrolyte was denoted as LBT-x (x represents the molar ratio of TTE to LB-3). As a comparison, LB-3 and LBT-1 without FEC additive were denoted as LB-3 (without FEC) and LBT-1 (without FEC). All electrolytes were prepared in an Ar-filled glove box with the H_2_O/O_2_ concentration below 0.2 ppm.

### Characterization

The viscosity of the as-prepared electrolytes was measured with an A&D SV-1A vibro-viscometer (A&D Co., Ltd, Tokyo, Japan) at 25 °C. The ionic conductivity of amide-based electrolytes was tested on the conductivity meter (Leici 308F, Shanghai) at 25 °C. Thermogravimetric analysis (TGA) was performed from room temperature to 600 ℃ with a heating rate of 10 ℃ min^−1^ under nitrogen atmosphere. Fourier transform infrared spectroscopy (FTIR) was tested by the IRTracer-100 spectrometer (Shimadzu, Japan) at room temperature. The Raman spectra were carried out by using a Raman spectrometer (Horiba, LabRAM HR800, USA). The morphology of Li deposited on Cu substrate after 10 cycles in Li||Cu cells was observed by FE-SEM (HITACHI, SU8010, Japan). In situ optical microscope was characterized by in situ cell module under an optical microscope (Weiscope, WSP300D, China). X-ray photoelectron spectroscopy (XPS) was performed on a PHI 5000 VersaProbe II spectrometer with monochromatic Al Kα X-ray radiation.

The LiMn_2_O_4_ (LMO) and LiFePO_4_ (LFP) cathodes were prepared by a conventional slurry coating method. The LMO/LFP active materials, carbon black and polyvinylidene fluoride were dispersed well in a FA25 superfine homogenizer in a mass ratio of 80:10:10, with N-methyl-2-pyrrolidone as the dispersant. The active materials mass loading of the cathode was 1.5–2.0 mg cm^−2^. Li chips were used as the anode and GF membranes (Whatman GF/A) as the separator. The oxidative stability of the electrolyte was tested by Li||SS cells from open circuit voltage to 6 V at a sweep rate of 1 mV s^−1^. The current response with different electrolytes was tested by CV in Li||Cu cell from -0.3 to 0.6 V. The charge and discharge tests were performed on the NEWARE BTS-51 battery test system at different temperatures with the voltage range of 2.5–4.2 V for LFP and 3.0–4.4 V for LMO. C rate is defined according to the theoretical capacities of LFP (170 mAh g^−1^) and LMO (117 mAh g^−1^). The electrochemical impedance spectroscopy (EIS) measurements were tested in the frequency range from 10^6^ to 10^–2^ Hz with a voltage amplitude of 10 mV. Cyclic voltammetry (CV), linear sweep Voltammetry (LSV) and EIS data were collected on a CHI 760E (CH, Shanghai, China) electrochemical workstation.

### Computational Details

All the all-atom MD simulations were based on a general AMBER force field [36] with the RESP charges [37] and were carried out using the Gromacs-4.6.7 software package [38]. The system is a relaxed liquid configuration at 298 K. The total run time was 10 ns NPT for the equilibrium MD simulation. We used the relaxed system as a starting configuration. As it is prior to system relaxation MD, energy minimization was carried out with a composite protocol of steepest descent using termination gradients of 100. The Nose´–Hoover thermostat [39] was used to maintain the equilibrium temperature at 298 K and periodic boundary conditions were imposed on all three dimensions. The particle mesh Ewald method [40, 41] was used to compute long-range electrostatics within a relative tolerance of 1 × 10^–6^. A cutoff distance of 1 nm was applied to real-space Ewald interactions. The same value was used for van der Waals interactions. The LINCS algorithm [42] was applied to constrain bond lengths of hydrogen atoms. A leapfrog algorithm [43] was used with a time step of 2 fs. The molecular structure optimization was performed using Gaussian 03 program package with B3LYP/6-311G** level [44], and each molecular was checked to have minimum energy without imaginary frequency. Electrostatic potential (ESP) [45] analysis and reduced density gradient (RDG) [46] analysis were conducted using Multiwfn [47] and Winvmd [48].

## Results and Discussion

### New Amide-based Electrolyte and Physicochemical Properties

LiTFSI and butyrolactam (BL) can form homogenous and transparent liquids (LB) at ambient temperature when molar ratio is predetermined from 1:2 to 1:4 (denoted as LB-x, x = 2, 3 and 4). Adding TTE to the LB-3 electrolyte (denoted as LBT-x; x = 0, 0.5, 1 and 1.5) can reduce the lithium salt concentration and viscosity, while increasing the ion conductivity. The physical properties of the LB and LBT electrolytes with different molar ratios are shown in Fig. S1. Of all the LB samples, the LB-3 electrolyte with a molar ratio of 1:3 shows the highest ionic conductivity (0.43 mS cm^−1^ at room temperature) and relatively low viscosity (238.1 mPa s). Taking the high ionic conductivity into consideration, we chose LB-3 for the further experiments. The viscosity of LBT-1 reduces to 29.5 mPa s, while the ion conductivity (1.25 mS cm^−1^) increases to three times as that of the pure LB-3. The melting temperature of LBT-0 is 1.7 ℃, which is lower than that of individual LiTFSI (~ 234 ℃) or BL (~ 23 ℃). The temperature drops to -16.2 ℃ for LBT-1 with the addition of TTE (Fig. S2).

**Fig. 1:**
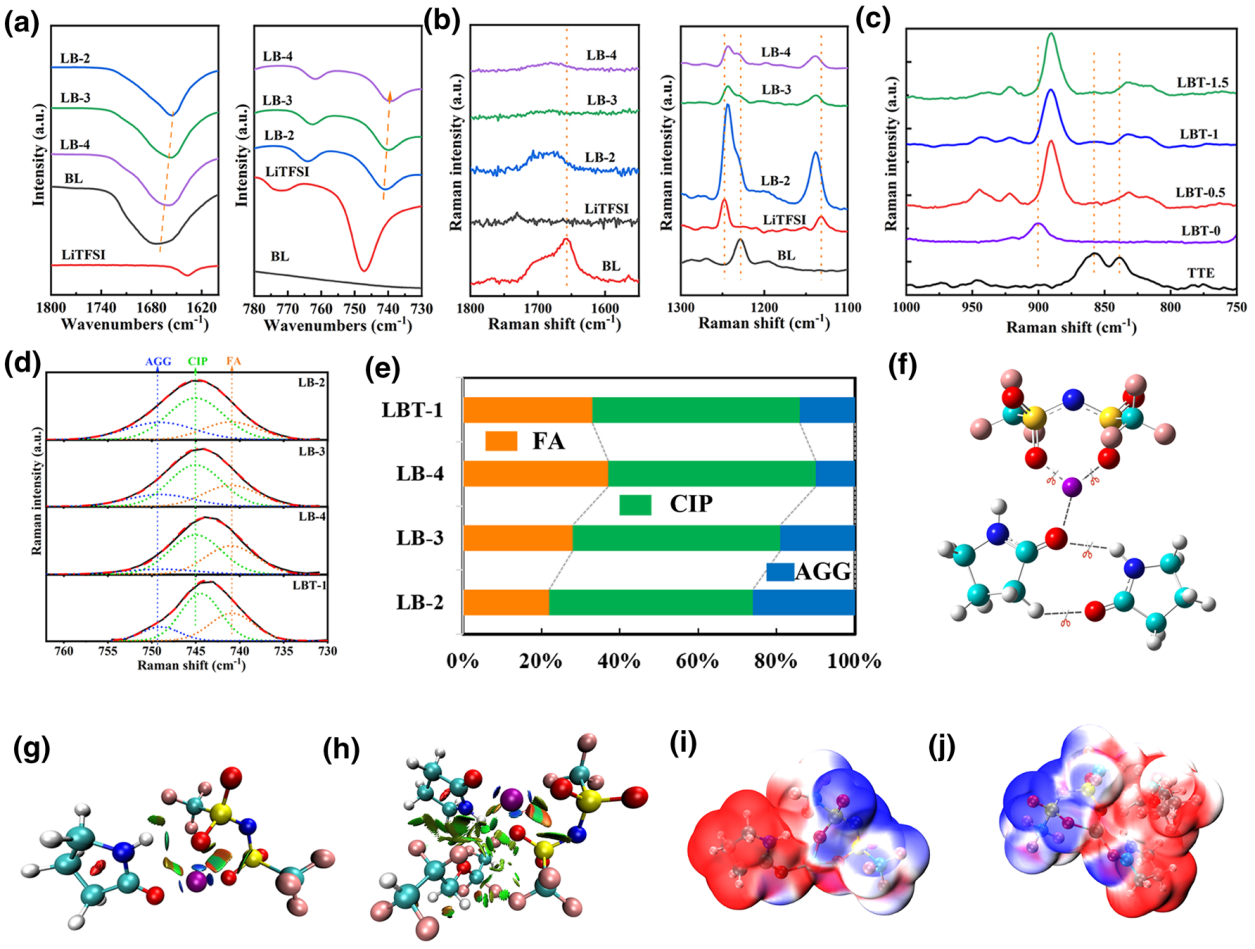
**a** FTIR spectra of LB electrolytes at different compositions. Raman spectra of **b** LB electrolytes and **c** LBT electrolytes at different compositions. **d** Fitted Raman spectra of LB electrolytes and LBT-1 in the range of 730–760 cm^−1^. The experimental spectra and fitted curves are denoted by the solid and dashed lines, respectively. **e** Solvate species distribution in LB electrolytes and LBT-1 determined by fitted Raman spectra. **f** Schematic diagram of LiTFSI and BL forming a liquid. Gradient isosurfaces (s = 0.5 au) for **i** LiTFSI-BL and **j** LiTFSI-BL-TTE. ESPs mapped on electron total density for **k** LiTFSI-BL and **l** LiTFSI-BL-TTE

The good thermal stability of the electrolyte can ensure the battery safety when a short circuit or thermal runaway occurs during operation. The thermal behaviors of LB and LBT electrolytes with different compositions were studied by TGA (Fig. S3). The onset temperature for degradation of LB electrolytes is over 160 ℃. Furthermore, the weight losses are only about 1.2% (LB-2), 1.3% (LB-3) and 1.1% (LB-4) after heating at 100 °C, demonstrating the thermal stability of LB electrolytes in the operating temperature region. In contrast, pure BL decomposes from 50 ℃ and suffers a 78% weight loss after heating to 160 ℃, implying that there is an interaction between BL and LiTFSI, which alleviates the decomposition of BL. And there is a certain weight loss before 100 ℃ for LBT electrolytes, which should be ascribed to the volatile characteristics of TTE. Therefore, the good thermal stability of LBT electrolytes allows the battery to be operated at higher temperatures. Figure S3c shows the weight loss of TTE in pure TTE and LBT-1 electrolyte at 30 ℃, and the pure TTE undergoes a completely weight loss because of evaporation within 6 min. In contrast, the weight loss of TTE in LBT-1 maintains for 30 min because TTE has weak interaction with LB electrolyte, which suppresses the evaporation of TTE. This result is consistent with the DFT calculation (Figs. 1h and S10).

### Solution Structure Analysis of LB and LBT Electrolyte

FTIR spectrum and Raman spectra were used to analyze the formation mechanism of amide-based electrolyte. For LB electrolytes, the FTIR bands at 1670 cm^−1^ assigned to C = O of BL and 747 cm^−1^ assigned to TFSI^−^ shift to 1657 and 739 cm^−1^, respectively, after solution formation (Fig. 1a), which resulting from the strong coordination interaction between Li^+^ and C = O of BL [49]. Upon introduction of LiTFSI, the band at 3230 cm^−1^, originating from BL’s NH stretching vibration, almost disappears (Fig. S4), indicating that BL does not exist as free solvent molecules [50]. Similar results can be also obtained from Raman spectra (Fig. 1b). Two obvious spectra changes appear from 3275 to 3396 cm^−1^ and from 1658 to 1681 cm^−1^, corresponding to NH stretching and C = O stretching of BL in the composite, respectively. The bands at 1248 and 1131 cm^−1^ are attributed to SO_2_ stretching and CF_3_ symmetric stretching of solid LiTFSI, respectively [51], and they shift to 1243 and 1138 cm^−1^ upon the addition of BL into LiTFSI. These results reveal that the spectral bands associated with the C = O and NH vibration of BL and the SO_2_ and CF_3_ vibration of LiTFSI change significantly when a homogeneous liquid is formed due to Li^+^ cations coordinated C = O of BL while NH interacts with SO_2_ group of TFSI^−^. The DFT optimized structure also confirms the interaction between -NH and S = O of TFSI^−^ (Fig. S9). The intermolecular interactions between LiTFSI and BL jointly weaken the individual bonds of original compositions and promote the separation of cations and anions, resulting in liquid state [50], as illustrated in Fig. 1f. For LBT electrolytes, the IR bands at 1180 cm^−1^ of TFSI^−^ and 1656 cm^−1^ of BL slightly change (Fig. S5). Figure 1c shows that two characteristic Raman peaks at 858 and 838 cm^−1^ assigned to TTE shift to 832 and 817 cm^−1^, respectively [52], and the band of BL shift from 900 to 890 cm^−1^ when introducing TTE into LB electrolyte (Fig. S6), owing to the weak interaction between TTE and LB.

It has been confirmed that the Raman peaks at around 720–760 cm^−1^ can be attributed to the free and Li-bond TFSI anion [53, 54]. As shown in Fig. 1d, the peaks at 741, 745 and 749 cm^−1^ corresponding to free anions (FA, TFSI^−^ coordinating 0 Li^+^), contact ion pairs (CIP, TFSI^−^ coordinating 1 Li^+^) and aggregate clusters (AGG, TFSI^−^ coordinating 2 Li^+^ or more Li^+^), respectively. The decrease in the lithium salt concentration with the increase in the BL leads to a decrease in the proportion of AGG while the FA content increases (Fig. 1e). Furthermore, the existence of TTE in BL electrolyte can also result in an increase in the proportion of free TFSI^−^, which indicates that TTE is beneficial to the dissociation of Li^+^ and TFSI^−^.

**Fig. 2:**
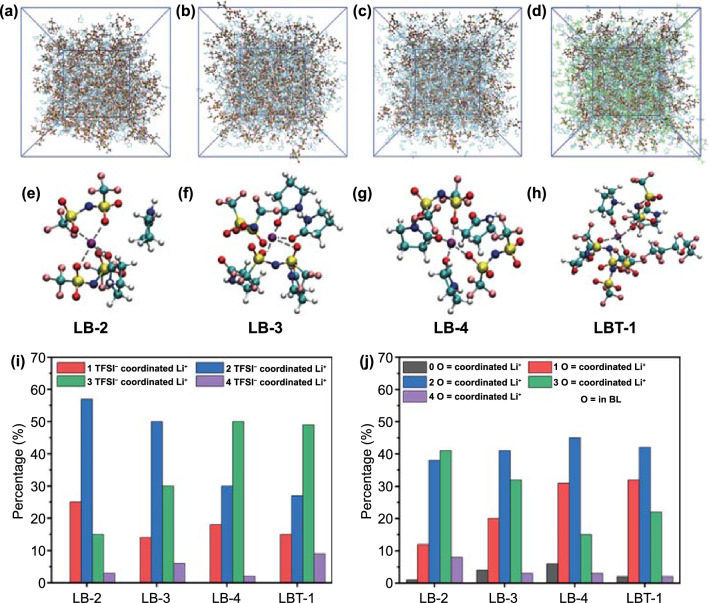
AIMD simulation snapshots of **a** LB-2, **b** LB-3, **c** LB-4 and **d** LBT-1 at 298 K. Ball and stick model stand for LiTFSI, while BL (blue) and TTE (green) are presented by wireframes. Representative Li^+^ coordination structure in **e** LB-2, **f** LB-3, **g** LB-4 and **h** LBT-1. Percentage of Li^+^ coordination structure in different electrolytes based on MD simulation. **i** Percentage of Li^+^ cations coordinated with different number of TFSI^−^ anions in LB-2, LB-3, LB-4 and LBT-1. **j** Percentage of Li^+^ cations coordinated with different number of BL molecules in LB-2, LB-3, LB-4 and LBT-1(O = represents the O atom in BL)

MD simulation was carried out to further understand the solvation structure of amide-based electrolytes and the effect of introducing TTE. Figure 2a–d shows the snapshots of LB-2, LB-3, LB-4 and LBT-1. The results of radial distribution function and coordination numbers for simulated electrolytes are demonstrated in Fig. S7. Two sharp peaks of Li-O_BL_ and Li-O_TFSI_ are observed at 1.97 and 2.02 Å for all studied electrolytes. This indicates that Li-O_BL_ and Li-O_TFSI_ are used as first coordinated shell (2.80 Å within Li^+^ cations). And Li-N_TFSI_, Li-N_BL_ and Li-O_TTE_ are far away from Li^+^ about 4 Å, which means that they are barely coordinated with Li^+^. With the increase in the BL content and the introduction of TTE, the coordination number of Li^+^ and TFSI^-^ decreases, which is conducive to the dissociation of Li^+^ and TFSI^-^ (Table S1). The molecular structures of LiTFSI, BL and TTE and corresponding atomic charge for MD simulations are shown in Fig. S8. The detailed results obtained from MD simulations are listed in Tables S1.

The statistics of MD simulation are shown in Fig. 2 and Table S1. In LB-2 and LB-3 electrolytes, Li^+^ tends to coordinate with two TFSI^−^ anion (two O atoms from two SO_2_ groups of TFSI^−^ anion). When the content of BL is increased for LB-4 and TTE is added for LBT-1, Li^+^ tends to coordinate with 3 TFSI^−^ anion (only one O atom from one SO_2_ group of TFSI^−^ anion). This result indicates that BL can facilitate the dissociation between Li^+^ and TFSI^−^ due to the strong coordination between Li^+^ and O = of BL. Meanwhile, the addition of TTE into LB electrolytes can also achieve the same result. This is a mutual verification with the peak fitting results of Raman characterizations.

DFT was performed to further investigate the coordination structure between LiTFSI and BL, and the optimized structures of different numbers of LiTFSI and BL are shown in Fig. S9. The energy is the lowest when Li^+^ is coordinated to two oxygen atoms on different -SO_2_ groups on the central nitrogen. The preference for 5-coordinate Li^+^ can be seen from the optimization results when one Li^+^/TFSI^−^ ion pairs with BL molecules from one to four. The optimized structure of LB-2 with four coordinates still allows more BL molecules to coordinate with the Li^+^ cation. With the introduction of BL molecules, the distance between Li^+^ and the sulfonyl oxygen atom of TFSI^−^ increases. Furthermore, Li^+^ coordinates with only one oxygen atom of one sulfonyl group with the number of BL above two. These results indicate that the strong coordination between BL and Li^+^ promotes the dissociation between Li^+^ and TFSI^−^, which is consistent with the MD simulations results. Reduced density gradient (RDG) analysis is an effective method for visually characterizing the regions and types of molecular weak interactions, including the van der Waals forces, hydrogen bonds and spatial repulsion [55]. As displayed in Fig. 1g, h and S10, a strong coordination bond exists between Li^+^ and the oxygen atom of the carbonyl group and sulfonyl groups. In addition, there are also significant van der Waals interaction between BL and TFSI^−^ anion, similar to the DFT results. Figure 1i, j and **S11** reveal the electron density of LB system by ESP analysis. The mutual attraction between the negatively electronegativity region of BL around the oxygen atoms and the positively charged regions around Li^+^ leads to the formation of a stable structure [56].

The interfacial compatibility of lithium metal and electrolyte is further investigated by symmetric Li||Li cells. Figure S12 shows the cycle performance of Li||Li symmetric cells under 0.2 mA cm^−2^. The cell with LBT-0 electrolyte shows obvious voltage fluctuation after 50 h and short circuit after 230 h; in contrast, the LBT-1 electrolyte can maintain stable cycling for more than 400 h, indicating LBT-1 electrolyte has better compatibility with lithium metal and more stable Li/electrolyte interface. The Coulomb efficiency was further examined by electrochemical testing of Li||Cu cells (Fig. S13). The 1 M LiPF_6_ EC-DEC electrolyte shows average CE of only 76.9%; in contrast, LBT-1 electrolyte has a better CE of 86.6%.

The stable electrochemical windows of all as-prepared electrolytes were tested using linear scanning voltammetry (LSV). As shown in Fig. S14, the anodic oxidation potential of all electrolytes is up to 4.7 V defined at 10 μA cm^−2^, which can be compatible with electrode materials working at a relatively high voltage. Furthermore, the influence of introducing TTE into LB electrolytes on the electrochemical window can be ignored. The current response in LBT-1 increases significantly during Li plating/stripping on Cu electrode, indicating the rapid Li^+^ transport and more reversible reaction kinetics (Fig. S15) [57].

### Spherical Li Deposition and In situ Observation of Li Plating

**Fig. 3:**
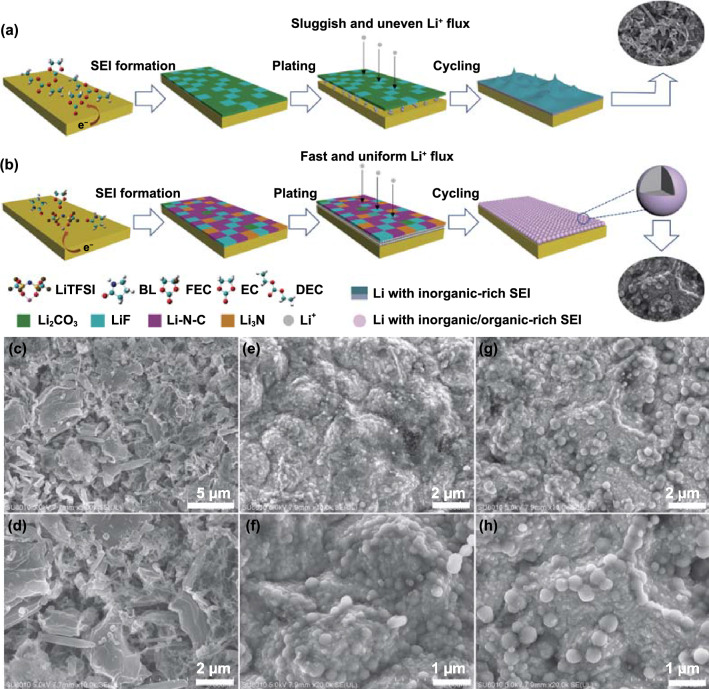
Schematic illustration of the Li plating behavior with different electrolytes: **a** 1 M LiPF_6_ in EC/DEC and **b** amide-based electrolytes. SEM images of Li metal after plating on Cu foil at 0.2 mA cm^−2^ with a capacity of 0.4 mAh cm^−2^ in different electrolytes: **c-d** 1 M LiPF_6_ in EC/DEC (1:1, v/v), **e–f** LBT-0 and **g-h** LBT-1

The morphology of Li deposition on Cu substrate in different electrolytes is presented in Fig. 3. In conventional carbonate-based electrolyte (1 M LiPF_6_ in EC/DEC), a large number of irregular Li dendrites can be clearly observed. The needlelike dendritic structure at the tip may penetrate through the separator and cause an internal short circuit, leading to serious safety risks. The growth of dendrites in carbonate-based electrolyte is mainly attributed to the low Li^+^ conductivity and uneven Li^+^ flux of SEI film formed by conventional organic solvents (Fig. 3a, c, d). On the contrary, the spherical Li deposition occurred in both LBT-0 and LBT-1 electrolytes. This result indicates a uniform and stable SEI film formed with high Li^+^ conductivity and uniform spatial distribution of Li^+^, resulting in spherical lithium deposition (Fig. 3b, g, h).

**Fig. 4:**
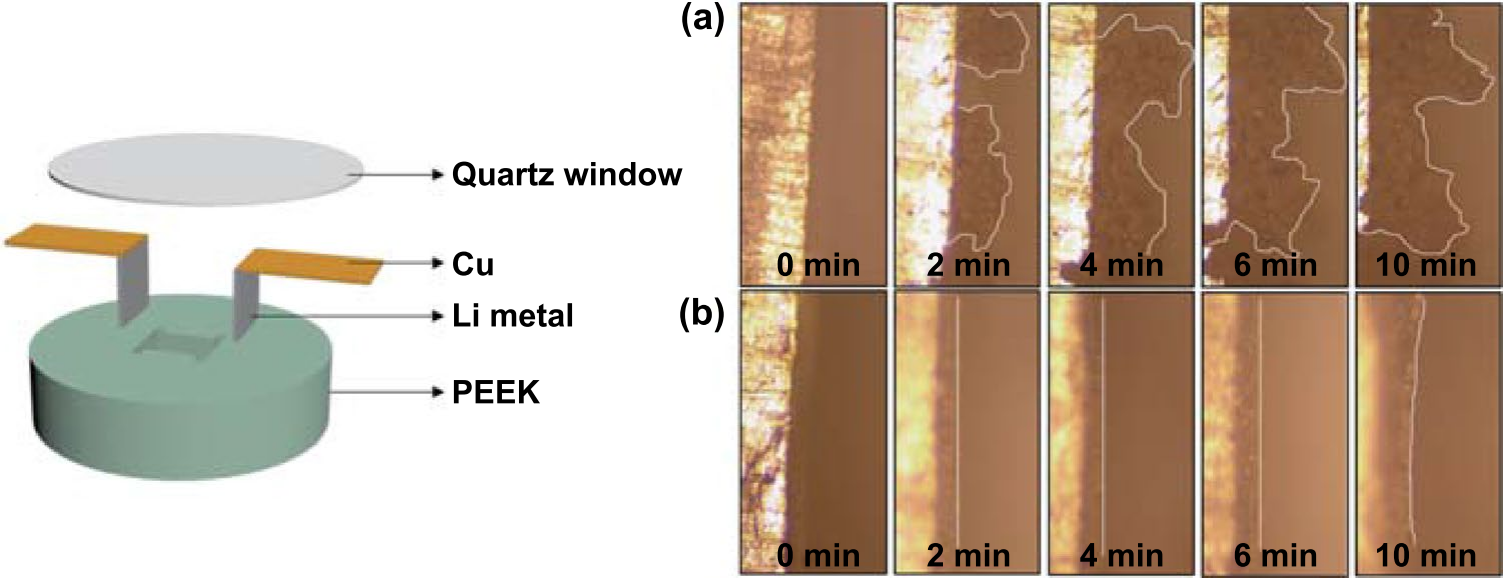
In situ optical microscopy observations of Li metal deposition with different electrolytes at the current density of 2 mA cm^−2^: **a** 1 M LiPF_6_ in EC/DEC (1:1 v/v) and **b** LBT-1

In order to visualize the morphology evolution of Li deposition at the electrolyte/electrode interface, in situ optical microscope was performed on the cells with different electrolytes. As shown in Fig. 4, the pristine Li electrode was smooth and flat before Li plating. For the carbonate-based electrolyte, a mosslike Li dendrites appeared as early as 2 min after Li plating, and the growth of Li dendrites boomingly evolved as time went on. In comparison, a dense and more uniform Li deposition with almost no dendritic structure was obtained with LBT-1 electrolyte. This observation indicates that LBT-1 electrolyte can effectively suppress the formation of Li dendrites and is desirable for the practical application of lithium metal batteries. The dynamic morphology evolution of Li deposition is consistent with SEM results.

### Chemical Composition Analysis of SEI Layer

**Fig. 5:**
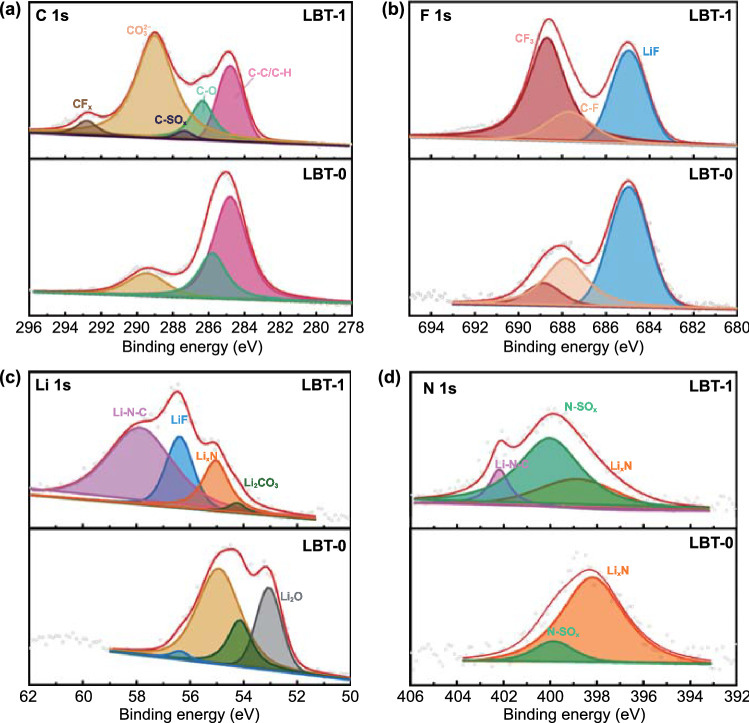
XPS spectra of **a** C 1s, **b** F 1s, **c** Li 1s, **d** N 1s of the Li anodes after 10 cycles in LBT-0 and LBT-1 electrolytes

Chemical compositions of the SEI surface were further investigated on the cycled Li metal anodes by X-ray photoelectron spectroscopy (XPS) comparing amide-based electrolytes (Fig. 5) and conventional EC/DEC electrolyte (Fig. S16). The survey spectra are displayed in Fig. S17. In C 1s spectra, the signals of C–C/C–H (284.8 eV), C–O (286.3 eV) and CO_3_^2−^ (289.0 eV) can be detected in both LBT-0 and LBT-1 electrolytes [57]. In contrast, two additional peaks corresponding to C–SO_x_ (287.4 eV) [58] and C–F_x_ (292.8 eV) [59] appear with the introduction of TTE, probably resulting from the decomposition of TFSI^−^ anions. The signals of C–F_x_ (688.7 eV) are further confirmed from F 1s spectra, and the other peak can be assigned to LiF (685.0 eV), which indicates the preferred reduction of FEC [60]. The peaks of LiF and C–F can also be observed in EC/DEC electrolyte. For the Li 1s spectra of EC/DEC electrolyte, the peaks around 55.4 and 56.4 eV can be attributed to the inorganic species of Li_2_CO_3_ and LiF [61]. For LBT-0 and LBT-1 electrolytes, the signals of Li_2_CO_3_ (54.2 eV), Li_3_N (55.0 eV) and LiF (56.4 eV) can be detected [27, 62]. On the contrast, one more peak at 57.9 eV corresponds to organic species (such as Li–N–C) after the addition of TTE, originating from the decomposition of BL and LiTFSI [63]. This result demonstrates that TTE can regulate the chemical composition of SEI by affecting the solvation structure of electrolyte. The organic species Li–N–C and the inorganic species (including LiF and LiN) jointly dominate the SEI chemistry in LBT-1. These Li–N species can be further validated by N 1s spectra (Fig. 5d), where the signals of Li_3_N (398.6 eV), N–SO_x_ (400.0 eV) and Li–N–C (402.2 eV) can be clearly observed [63]. However, Fig. S16c reveals the absence of N species in EC/DEC electrolytes. In addition, the atomic content of F and N in the SEI film formed in LBT-1 is much higher than that in LBT-0 and EC/DEC electrolytes (Fig. S18). Consequently, the SEI film contains abundant inorganic species (LiF and Li_3_N) and organic species (C–N, S–N, and C–S) [29]. The inorganic/organic-rich SEI film possesses high ionic conductivity and high interface energy, which can synergistically regulate the nucleation of Li, contributing to the uniform and rapid lithium deposition and resulting in spherical Li deposition.

### Highly Stable LMBs with LiFePO_4_ (LFP) and LiMn_2_O_4_ (LMO) Cathodes

**Fig. 6:**
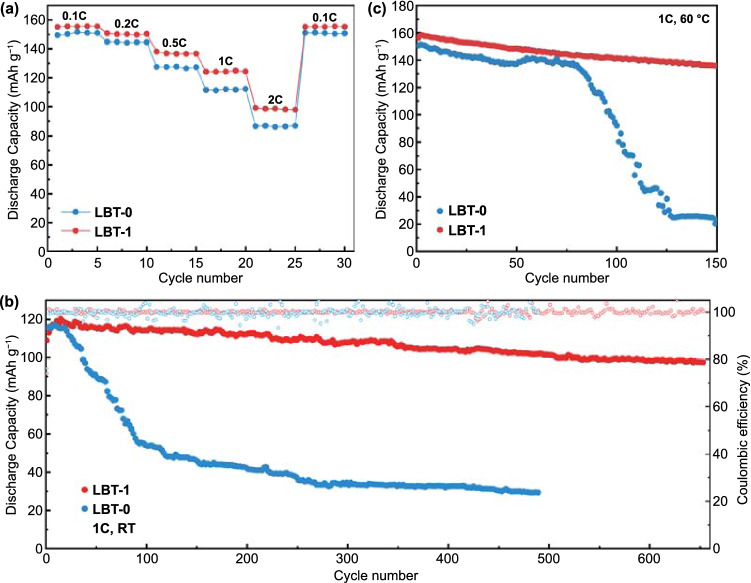
Performance of Li||LFP cell with different electrolytes: **a** rate performance at room temperature, **b** cycle performance at 1 C and room temperature and **c** cycle performance at 1 C and 60 ℃

**Fig. 7:**
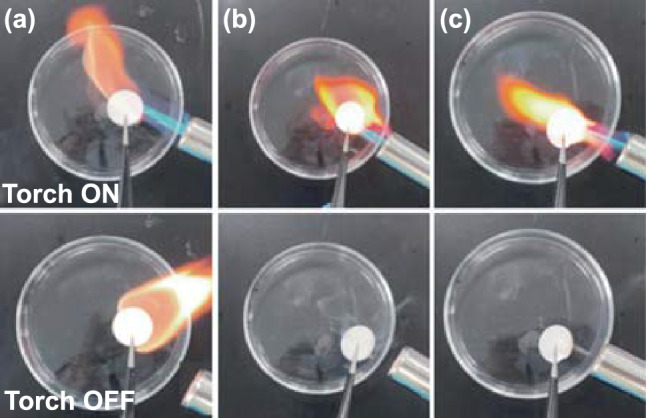
Photograph images of the flame tests of **a** 1 M LiPF_6_ EC/DEC, **b** LBT-0 and **c** LBT-1 with a torch turned on (up) and off (down)

The potential application and feasibility of amide-based electrolytes were further evaluated in LFP||Li and LMO||Li full cells. The rate performance of LFP||Li cell was evaluated in two electrolytes from 0.1 to 2 C (Fig. 6a). The LBT-1 electrolyte demonstrates better rate performance compared to the LBT-0 electrolyte. The specific capacities of LBT-1 electrolyte are 155.5, 150.6, 136.9, 124.8, 99.1 mAh g^−1^ at the current densities of 0.1, 0.2, 0.5, 1 and 2 C, respectively. Figure 6b displays that the long-term cycling performance of LBT-0 and LBT-1 electrolytes at 1 C and room temperature (~ 22 ℃). The cell with LBT-1 electrolyte exhibits outstanding cycling performance, remaining a capacity retention of 89% after 650 cycles. In contrast, the cell using LBT-0 electrolyte suffers from severe capacity fading, only remaining a capacity retention of 35% after 200 cycles. And the cell with carbonate electrolyte has a higher initial discharge capacity compared to the LBT electrolytes, but only maintains a capacity retention of 60% after 650 cycles at 1 C (Fig. S19a). Since TTE has a high boiling point (92 ℃) and LB electrolytes show superior thermal stability over 160 ℃, the LBT electrolytes with TTE also support operation at higher temperatures. As shown in Fig. 6c, the capacity of LFP with LBT-1 electrolyte still maintains 87% at 60 ℃ after 150 cycles. In comparison, the capacity of LBT-0 electrolyte declines sharply to 13% at 60 ℃. The introduction of TTE can reduce the viscosity, increase the conductivity and improve the compatibility with lithium metals simultaneously. The compatibility of amide-based electrolytes to the high-voltage LMO cathode was also evaluated in an LMO||Li full cell. As shown in Fig. S20, LBT-1 electrolyte exhibits an initial specific capacity of 106.6 mAh g^−1^ with 89% capacity retention after 100 cycles at 0.2 C from 3.0 to 4.4 V, which is better than LBT-0 electrolyte with an initial specific capacity of 90.2 mAh g^−1^. In comparison, there is only 66.2% capacity retention for LMO||Li cell using carbonate electrolyte at 0.2 C after 100 cycles (Fig. S19b). To clarify the role of FEC additive, the cycling performance of LB-3 (without FEC) and LBT-1 (without FEC) was compared (Fig. S21). The cycling performance of both LB-3 (without FEC) and LBT-1 (without FEC) decays rapidly, indicating that FEC as a SEI film-forming additive is critical for improving battery performance. These results indicate that both FEC and TTE contribute to outstanding cycling performance. The typical charge–discharge profiles of Li||LFP cell and Li||LMO cell at different cycles of amide-based electrolytes and carbonate electrolyte are shown in Figs. S22 and S23, which indicates that the polarization of the LBT-0 electrolyte increases sharply with increasing cycles. Figure S24 shows the voltage profiles of two electrolytes at various currents and potential gaps of the first cycle at 0.1 C, indicating a significant decrease in polarization of LBT-1. The lower polarization can be attributed to the lower viscosity and faster lithium-ion migration rate through the organic/inorganic-rich SEI due to the existence of Li–N–C, LiF and Li_3_N. The excellent performance of LBT-1 electrolyte should be attributed to its higher ionic conductivity, inorganic/organic-rich stable SEI, better kinetics and lower polarization. In addition, the dendrite-free Li deposition also contributes to its superior performance. Notably, both LBT-0 and LBT-1 electrolytes are nonflammable and offer good safety in case of battery fire or explosion. In contrast, commercial EC-DEC electrolytes are inherently flammable, which limits their applications in future batteries (Fig. 7).

## Conclusion

In summary, a new type of amide-based nonflammable electrolyte has been successfully designed to significantly improve the safety and recyclability of lithium metal batteries. The introduction of TTE reduces the viscosity while improving the interfacial compatibility with lithium metal. Furthermore, the in situ formation of inorganic/organic-rich SEI layer consisting of LiF, Li_3_N and Li–N–C enables the uniform and rapid deposition of Li^+^, resulting in spherical lithium deposition. As a result, a lithium metal battery with LFP cathode displays very reliable cyclability and achieves an 89% capacity retention after 650 cycles. The cells can also operate stably even at 60 °C. Notably, the amide-based electrolyte possesses excellent electrochemical stability (> 4.7 V), providing another option for the application of high-voltage electrode materials. We are convinced that this work will provide a facile and effective strategy to alleviate the critical issues of lithium metal anodes, providing a deep insight into the development of highly safe, dendrite-free and durable lithium metal batteries.

## Supplementary Information

Below is the link to the electronic supplementary material.

Supplementary file1 (PDF 1350 kb)
