# Supplementary material for: Safe and Stable Lithium Metal Batteries Enabled by an Amide-Based Electrolyte
Source: Nanomicro Lett. 2022 Jan 12;14:44. doi: 10.1007/s40820-021-00780-7 (PMC8753956; doi:10.1007/s40820-021-00780-7)
Supplement: Supplementary file 1 — Supplementary file1 (PDF 1350 kb) [file 40820_2021_780_MOESM1_ESM.pdf]

Nano-Micro Letters

Supporting Information for

Safe and Stable Lithium Metal Batteries Enabled by an Amide-Based Electrolyte

Wanbao Wu[1, 2], Yiyang Bo[1, 2], Deping Li[3], Yihong Liang[1, 2], Jichuan Zhang[4], MiaoMiao Cao[1, 2], Ruitian Guo[1, 2], Zhenye Zhu[3], Lijie Ci[3, *], Mingyu Li[1, 2] and Jiaheng Zhang[1, 2, *]

[1]Sauvage Laboratory for Smart Materials, Harbin Institute of Technology (Shenzhen), Shenzhen 518055, P. R. China

[2]Research Centre of Printed Flexible Electronics, School of Materials Science and Engineering, Harbin Institute of Technology (Shenzhen), Shenzhen 518055, P. R. China

[3]School of Materials Science and Engineering, Harbin Institute of Technology, Shenzhen 518055, P. R. China

[4]Department of Chemistry, University of Idaho, Moscow, ID 83844-2343, USA

*Corresponding authors. E-mail: cilijie@hit.edu.cn (Lijie Ci), zhangjiaheng@hit.edu.cn (Jiaheng Zhang)

Supplementary Figures and Table

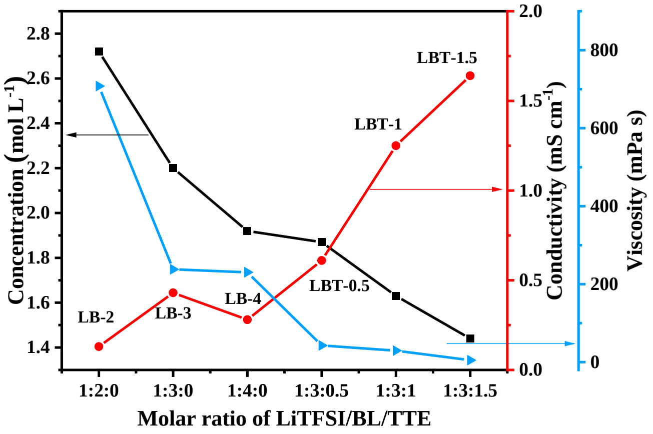

Fig. S1 Concentration, viscosity, and conductivity of LB and LBT electrolytes at different compositions

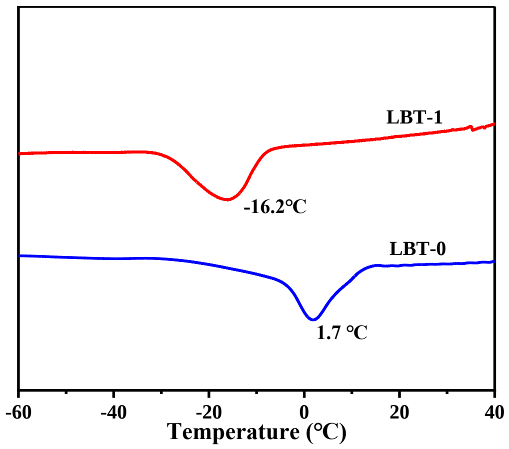

Fig. S2 DSC results for LBT-0 and LBT-1

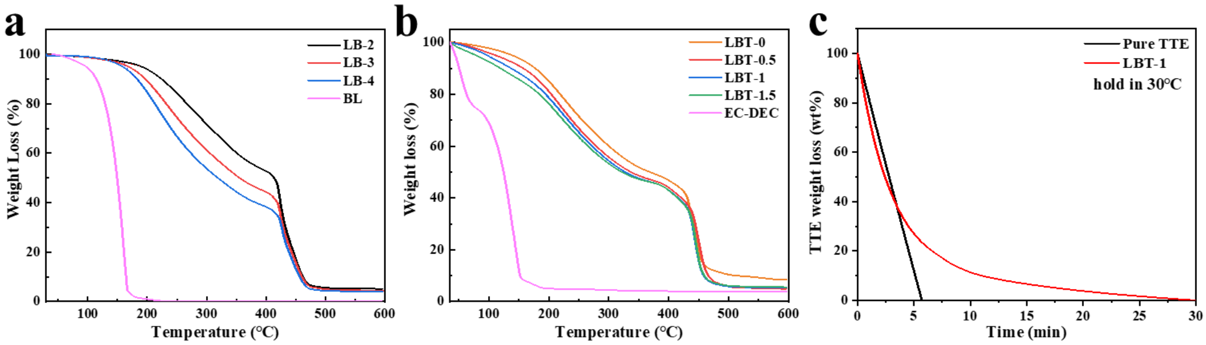

Fig. S3 Thermo gravimetric analysis (TGA) of (**a**) LB electrolytes and (**b**) LBT electrolytes with different molar ratios. (**c**) Isothermal TG curves of pure TTE and LBT-1 measured at 30 °C

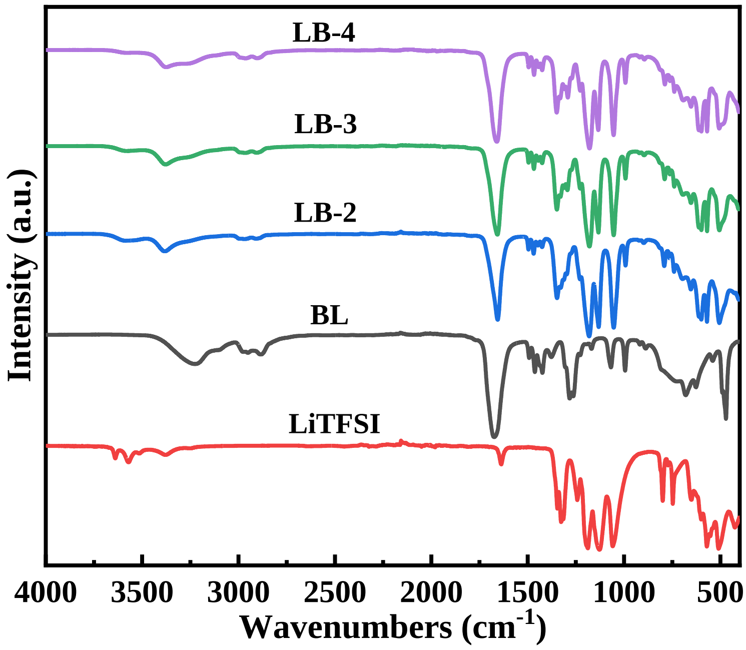

Fig. S4 FTIR spectra of LiTFSI, BL and LB electrolytes with different molar ratios (1:2−1:4)

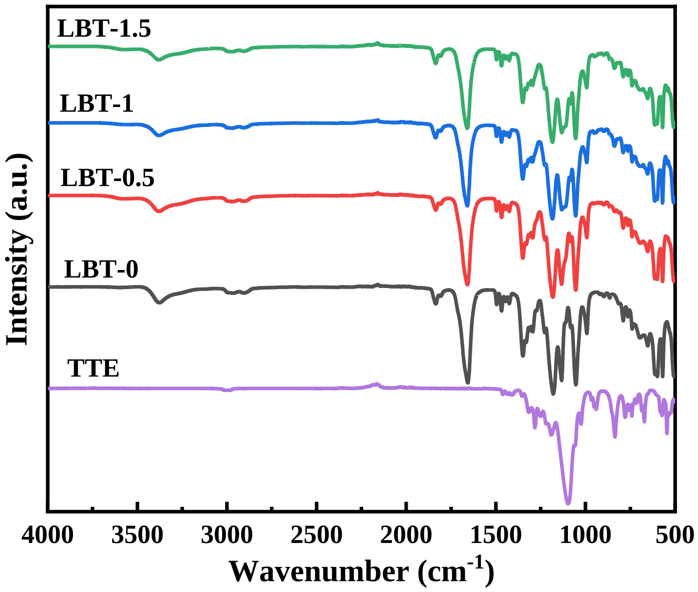

Fig. S5 FTIR spectra of TTE and LBT electrolytes with different molar ratios (1:3:0−1:3:1.5)

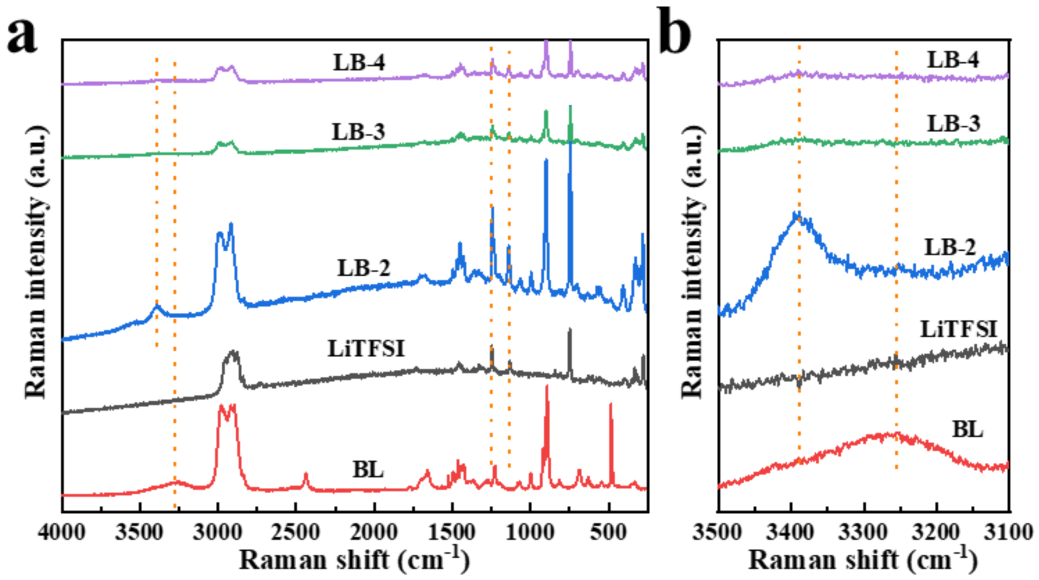

Fig. S6 Raman spectra of (**a**) BL, LiTFSI and LB electrolytes with different molar ratios (1:2−1:4), (**b-d**) Partial enlargement view of (**a**)

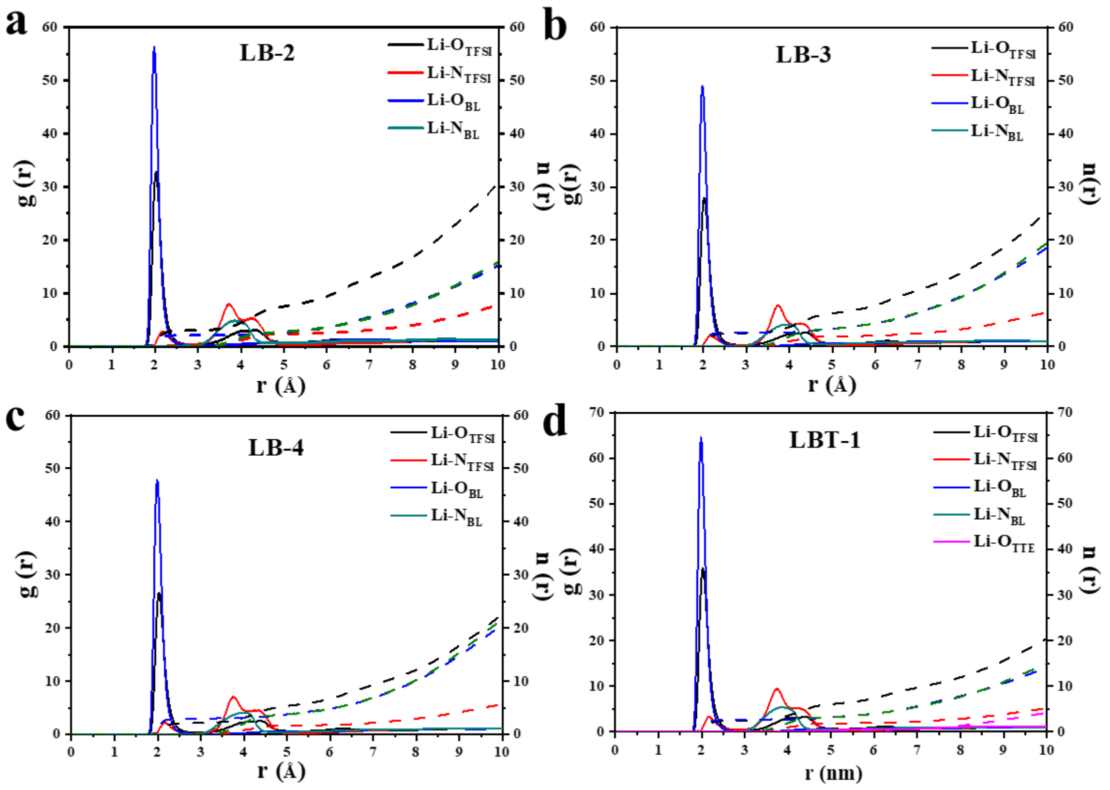

Fig. S7 Radial distribution function (g(r), solid lines) and coordination numbers (n(r)), dash lines) for (**a**) LB-2, (**b**) LB-3, (**c**) LB-4, and (**d**) LBT-1

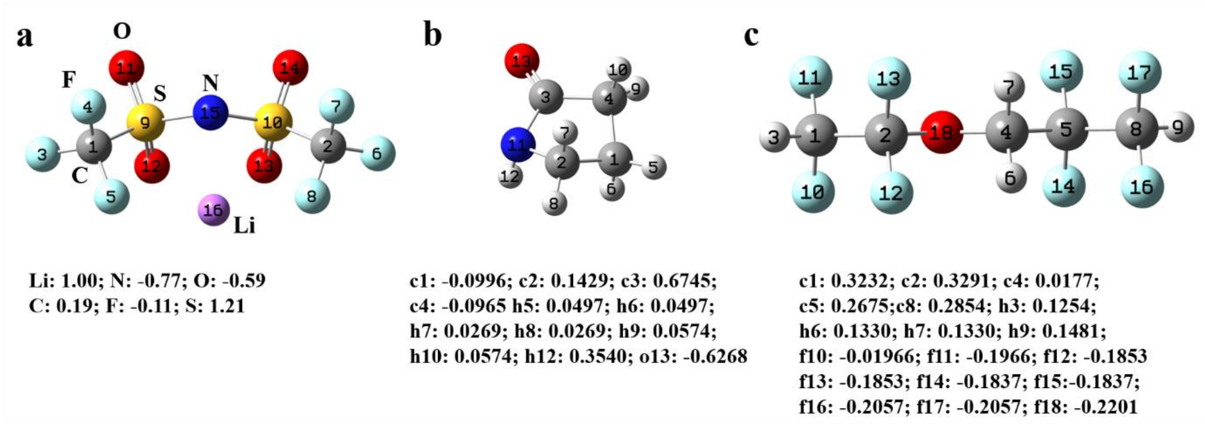

Fig. S8 Structures for (**a**) LiTFSI, (**b**) BL, and (**c**) TTE molecules. The atom charges for the MD simulations are listed below its structure.

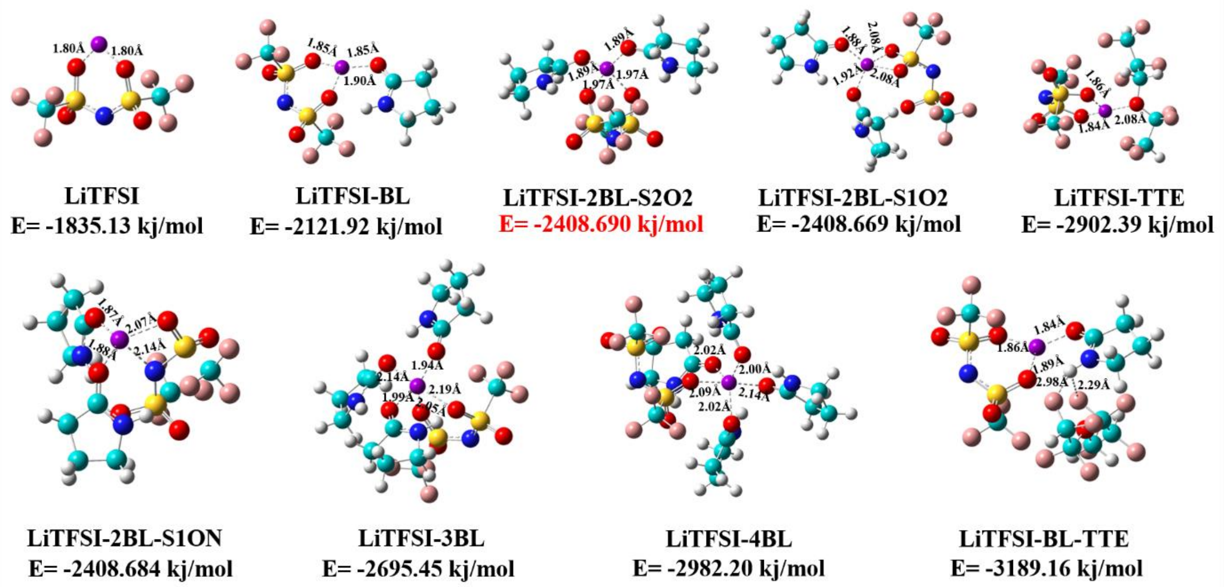

Fig. S9 DFT geometry optimization of the [LiTFSI(BL)n] and LiTFSI/BL/TTE complexes

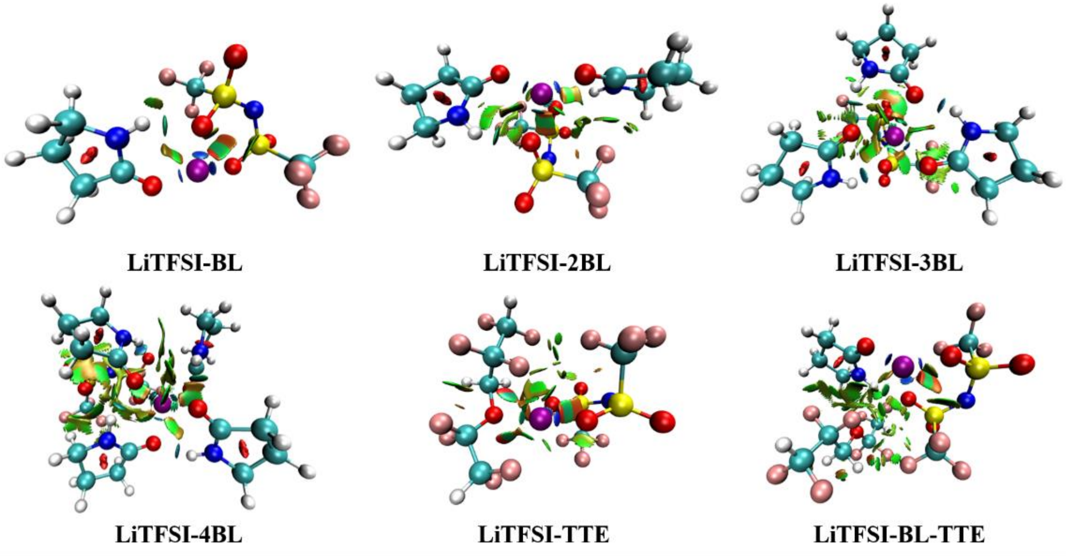

Fig. S10 Gradient isosurfaces (s=0.5 au) for [LiTFSI(BL)n] and LiTFSI/BL/TTE complexes. The surfaces are displayed in blue-green-red scale, ranging from -0.04 to 0.02 au

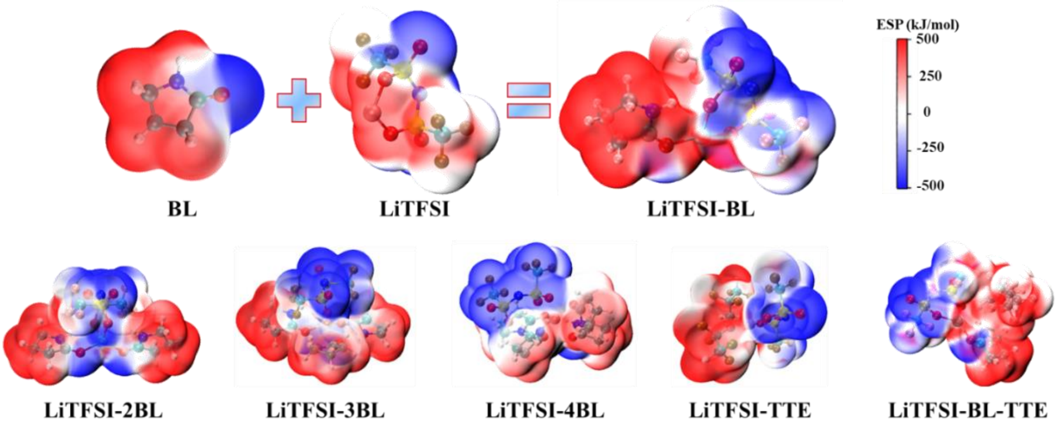

Fig. S11 ESPs mapped on electron total density for BL, LiTFSI, LiTFSI(BL)n (n = 1 to 4), LiTFSI-BL-TTE and LiTFSI-TTE species

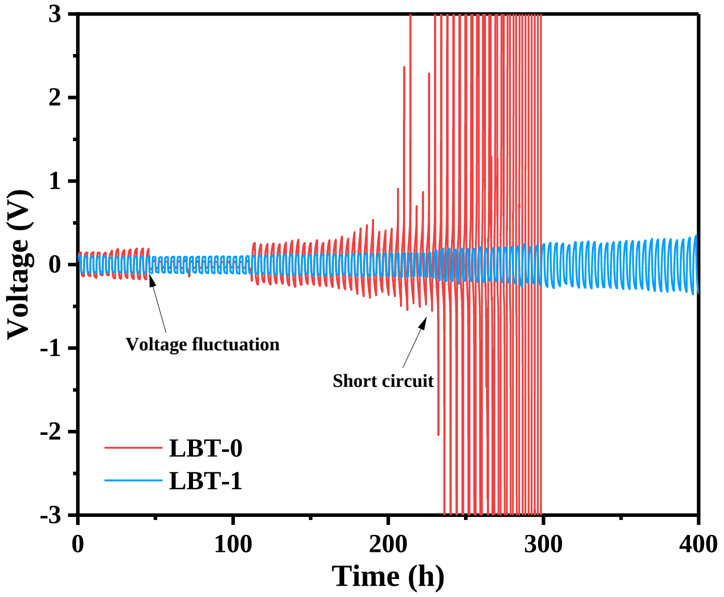

Fig. S12 Voltage responses of Li/Li symmetric cells in LBT-0 and LBT-1 electrolytes at 0.2 mA cm^{-2} and 0.4 mAh cm^{-2}

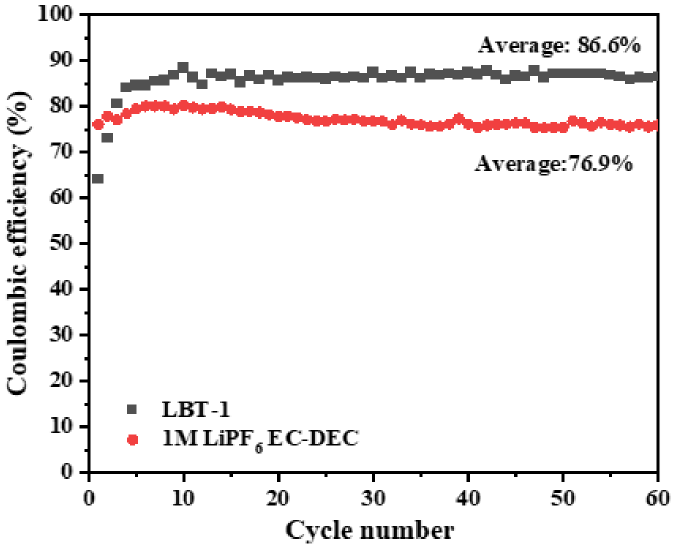

Fig. S13 CE of Li plating/stripping profiles in LBT-1 and 1M $LiPF_6$ EC-DEC electrolytes at a current density of 0.2 mA cm^{-2} and the capacity of 0.2 mAh cm^{-2}

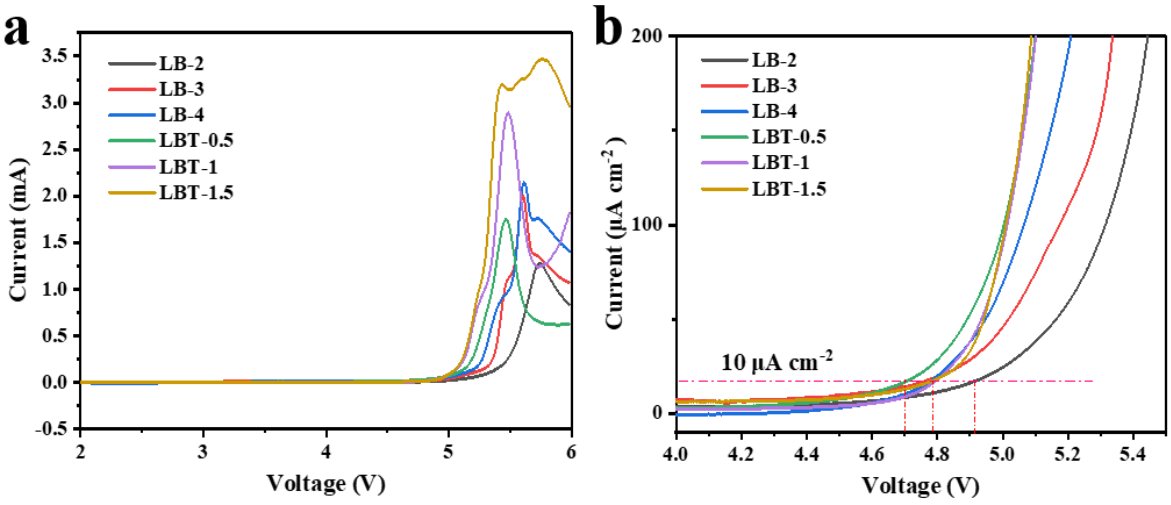

Fig. S14 Linear sweep voltammetry (LSV) curves of (**a**) different electrolytes and (**b**) partial enlarged view of (**a**)

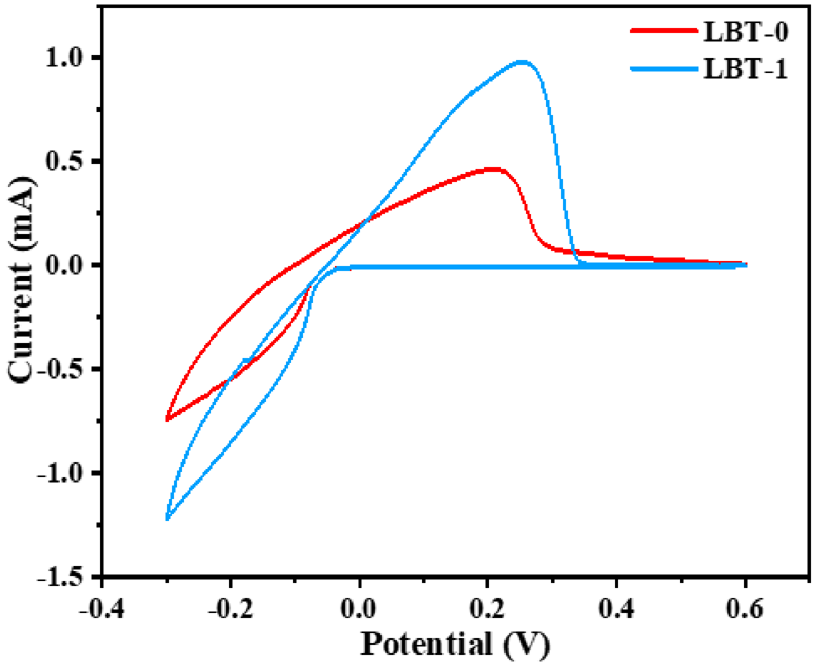

Fig. S15 CV curves of Li||Cu cells of electrolytes between -0.3 and 0.6 V at a scanning rate of 1 mV s^{-1}

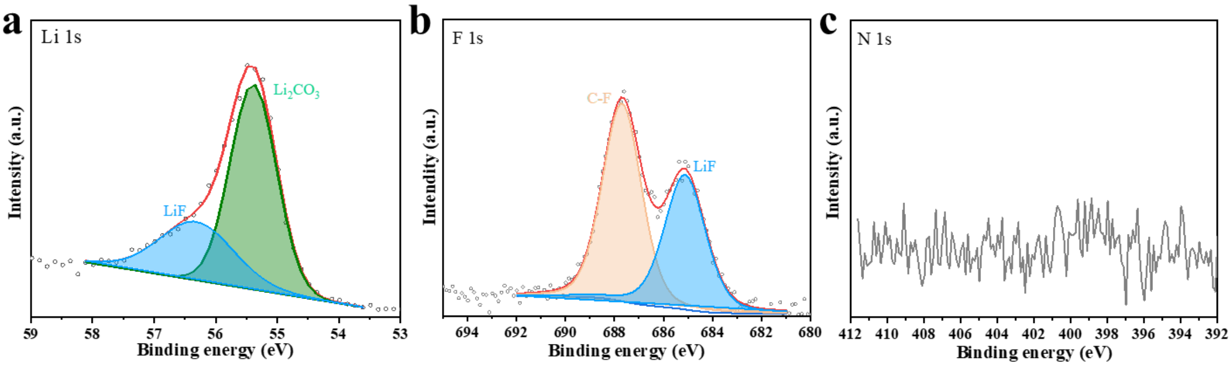

Fig. S16 XPS spectra of (**a**) Li 1s, (**b**) F 1s and (**c**) N 1s of Li anodes after 10 cycles in 1 M $LiPF_6$-EC-DEC electrolytes

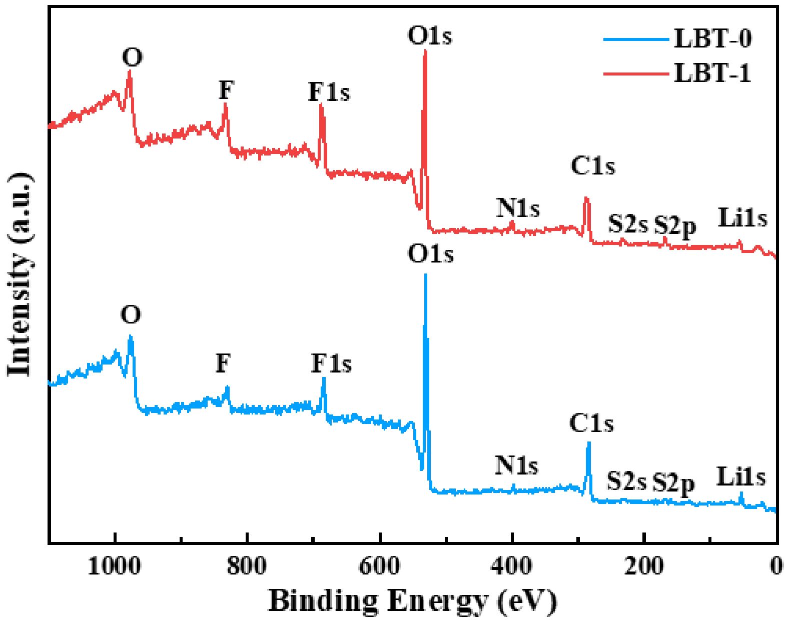

Fig. S17 Survey spectra of Li anodes after 10 cycles in LBT-0 and LBT-1

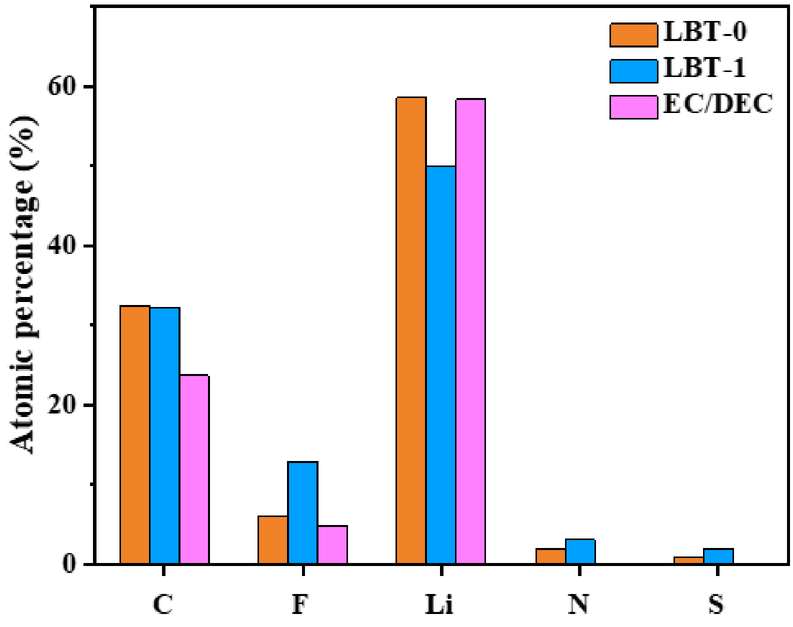

Fig. S18 Atomic percentage of different elements in different electrolytes

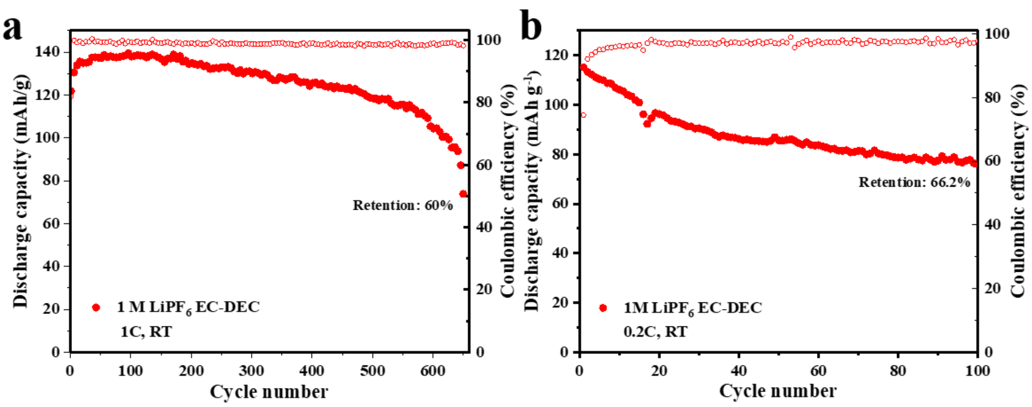

Fig. S19 Cycle performance of (**a**) LFP||Li cell at 1C and (**b**) Li||LMO cell at 0.2C with 1 M $LiPF_6$ EC-DEC

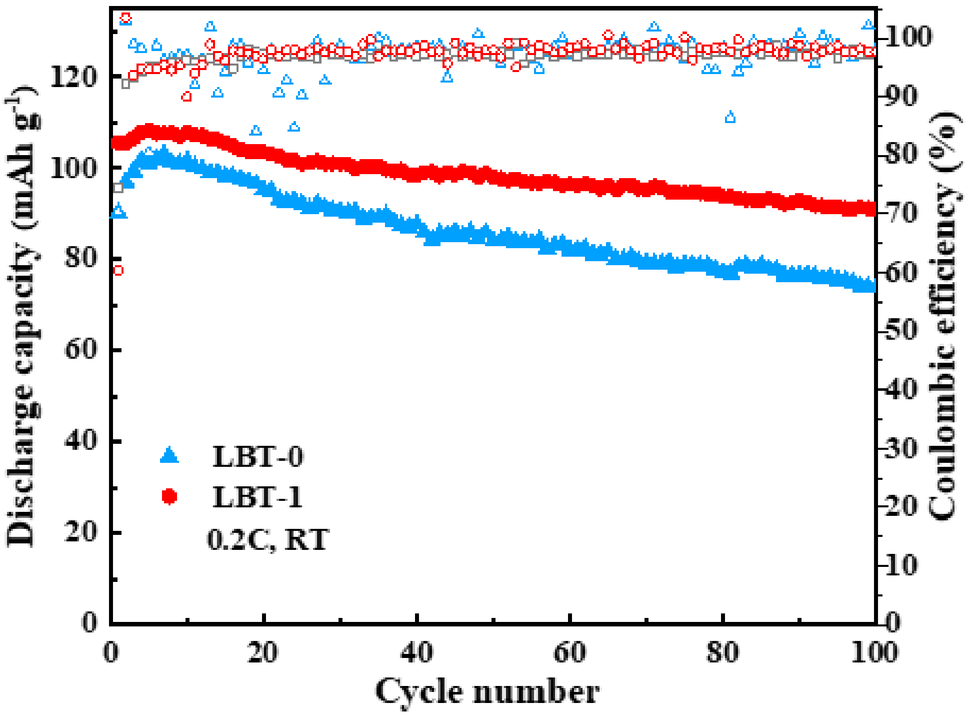

Fig. S20 Cycle performance of LMO||Li cell with different electrolytes at 0.2 C and room temperature

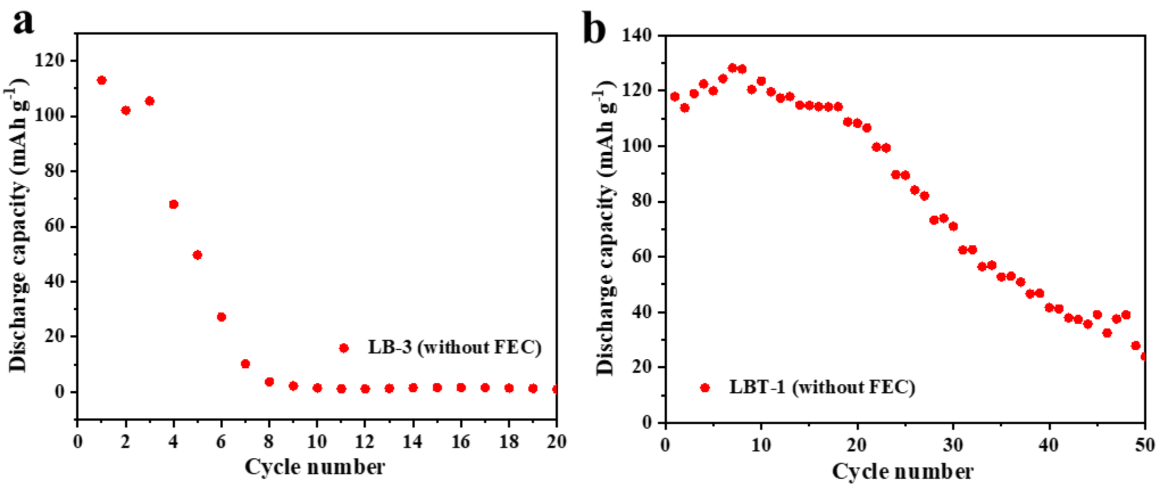

Fig. S21 Cycling performance at 1C and room temperature of (**a**) LB-3 (without FEC), (**b**) LBT-1 (without FEC)

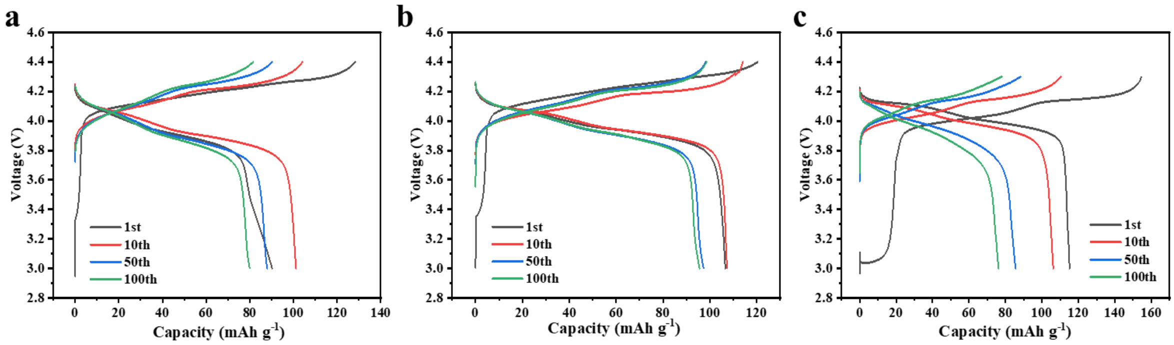

Fig. S22 Voltage curves of LMO||Li cells using (**a**) LBT-0, (**b**) LBT-1, and (**c**) 1 M $LiPF_6$ EC/DEC electrolyte at 0.2 C

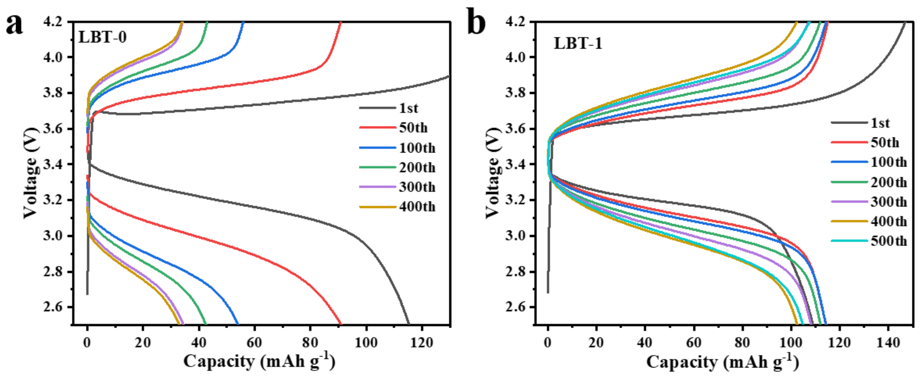

Fig. S23 Voltage curves of LFP||Li cells using (**a**) LBT-0 and (**b**) LBT-1 electrolyte under the voltage ranges of 2.5- 4.2 V at 1 C

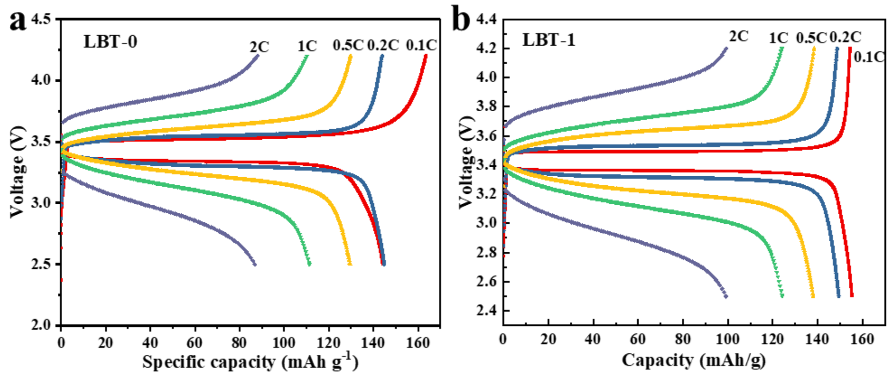

Fig. S24 Typical charge-discharge profiles of LFP||Li cell at different rate of (**a**) LBT-0 and (**b**) LBT-1

Table S1 MD simulation details for the LB-2, LB-3, LB-4, and LBT-1electrolytes

**Electrolyte**		**LB-2**	**LB-3**	**LB-4**	**LBT-1**
No. LiTFSI in box		400	400	400	400
No. LB in box		800	1200	1600	1200
No. TTE in box		-	-	-	400
Li coordination No.					
O= (within 2.80 Å of Li) in TFSI-		3.19	2.63	2.16	2.52
O= (within 2.80 Å of Li) in BL		2.14	2.62	2.98	2.53
-O- (within 2.80 Å of Li) in TTE					0.098
-N- (within 4.80 Å of Li) in TFSI		2.26	1.81	1.45	1.96
-N- (within 4.80 Å of Li) in BL		2.12	3.09	3.40	3.19
Li^+ within 2.80 Å of O= in BL	0	1	4	6	2
	1	12	20	31	32
	2	38	41	45	42
	3	41	32	15	22
	4	8	3	3	2
	5	0	0	0	0
Li^+ within 4.80 Å of N in TFSI	0	0	0	0	0
	1	25	14	18	15
	2	57	50	30	27
	3	15	30	50	49
	4	3	6	2	9
	5	0	0	0	0
